# Coevolving MAPK and PID phosphosites indicate an ancient environmental control of PIN auxin transporters in land plants

**DOI:** 10.1002/1873-3468.12929

**Published:** 2017-12-25

**Authors:** Magdalena Dory, Elizabeth Hatzimasoura, Brigitta M. Kállai, Szilvia K. Nagy, Katalin Jäger, Zsuzsanna Darula, Tímea V. Nádai, Tamás Mészáros, Enrique López‐Juez, Beáta Barnabás, Klaus Palme, László Bögre, Franck A. Ditengou, Róbert Dóczi

**Affiliations:** ^1^ Institute of Agriculture Centre for Agricultural Research Hungarian Academy of Sciences Martonvásár Hungary; ^2^ School of Biological Sciences and Centre for Systems and Synthetic Biology Royal Holloway, University of London Egham UK; ^3^ Department of Medical Chemistry Molecular Biology and Pathobiochemistry Semmelweis University Budapest Hungary; ^4^ Laboratory of Proteomics Research Biological Research Centre Hungarian Academy of Sciences Szeged Hungary; ^5^ Institute of Biology II University of Freiburg Germany; ^6^ BIOSS Centre for Biological Signalling Studies University of Freiburg Germany; ^7^ Centre for Biological Systems Analysis (ZBSA) University of Freiburg Germany

**Keywords:** Arabidopsis, MAP kinase, PIN, protein phosphorylation, signalling

## Abstract

Plant growth flexibly adapts to environmental conditions, implying cross‐talk between environmental signalling and developmental regulation. Here, we show that the PIN auxin efflux carrier family possesses three highly conserved putative mitogen‐activated protein kinase (MAPK) sites adjacent to the phosphorylation sites of the well‐characterised AGC kinase PINOID, which regulates the polar localisation of PINs and directional auxin transport, thereby underpinning organ growth. The conserved sites of PIN1 are phosphorylated *in vitro* by two environmentally activated MAPKs, MPK4 and MPK6. In contrast to AGC kinases, MAPK‐mediated phosphorylation of PIN1 at adjacent sites leads to a partial loss of the plasma membrane localisation of PIN1. MAPK‐mediated modulation of PIN trafficking may participate in environmental adjustment of plant growth.

## Abbreviations

**ER**, endoplasmic reticulum

**HL**, hydrophilic loop

**MAPK**, mitogen‐activated protein kinase

**MKK**, MAPK kinase

**PAT**, polar auxin transport

**PM**, plasma membrane

The mitogen‐activated protein kinase (MAPK) phosphorylation cascades are conserved signalling modules in all eukaryotes, consisting of three classes of enzymes, which are activated in a sequential phosphorylation cascade 1. Plant MAPKs have been mainly associated with stress signalling, but their role in developmental processes is increasingly evident 2, 3, 4, 5, 6. In the model plant Arabidopsis, genes encoding 20 MPKs and 10 MKKs (MAPK kinases) were identified, and both MPKs and MKKs are classified into four phylogenetic groups designated A–D 7. The best‐studied MAPKs represent group A (MPK3/6) and group B (MPK4). Both MPK6 and MPK4 are activated by environmental stress stimuli, for example, cold or the bacterial elicitor flagellin 8, 9, 10, but additionally both also participate in developmental processes. For example, MPK3/6 function in reproductive development 11, 12, 13, stomatal development 14, 15, cell division 16, 17, 18, 19 or photomorphogenesis 20. *mpk4* mutants are severely dwarfed 21 and the function of MPK4 in cytokinesis is well‐studied 22, 23, 24.

Such functional diversity and complexity of MAPK pathways imply complex substrate repertoires. Accordingly, human MAPKs phosphorylate a high number of functionally diverse substrates 25, 26. The knowledge on plant MAPK substrates is limited in comparison to mammalian organisms, nevertheless known substrate proteins represent a similar functional diversity: many are involved in defence while a number of recently identified substrates have developmental functions 12, 16, 20, 27, 28.

Plasma‐membrane‐localised PIN proteins are important in the establishment of polar auxin transport (PAT), a process generating auxin gradients, which are key determinants of organ formation and development. Plasma membrane (PM) localisation is generally associated with the presence of a long hydrophilic loop (HL) (PIN1/2/3/4/7), while short‐HL PINs (PIN5/8) are predominantly localised to the endoplasmic reticulum (ER) 29.

Auxin transporters are subject to post‐translational modification, and the characteristic polar membrane localisation of PIN is regulated by phosphorylation of PIN by the protein kinase PINOID (PID) and the related WAG1/2 kinases 30, 31, 32, 33, 34, 35. Moreover, not only the localisation but the auxin transporter activity of PINs are also regulated by PID and the D6 protein kinases 36, 37. In addition, it has been recently reported that PIN1 is phosphorylated on a poorly conserved S337 and that the MKK7‐MPK6 pathway regulates PIN1 polarity in the shoot stem 38. Identified phosphorylation sites of these kinases are all located within the long‐HL sequences, specific to PM‐localised PINs.

In this work, we set out to investigate three uncharacterised conserved MAPK phosphorylation sites in long‐HL PIN proteins adjacent to the similarly conserved PID sites. As both of these phosphosites can be traced back to the common ancestor of land plants, they might play a connected role in the regulation of PM‐localised PINs.

## Materials and methods

### Molecular cloning

MKK7 was amplified from genomic DNA (intronless gene) and was cloned into *pGEM‐T Easy* vector (Promega, Madison, WI, USA). The c‐myc epitope tag was introduced to generate N‐terminal epitope‐tagged derivatives. To generate a plant transformation vector the *myc:MKK7* fragment was first subcloned into the Gateway entry vector *pENTR4* (Invitrogen, Carlsbad, CA, USA). From *pENTR4*, the tagged gene version was moved into the expression vector *pER8GW*, a Gateway version of the β‐estradiol‐inducible expression vector pER8 39. The resulting transformation construct was designated *pER8GW:myc:MKK7* (*pER8:MKK7*).

The hydrophilic loop (HL: residues 156–482) of *PIN1* cDNA was amplified and cloned into *pGEM‐T Easy* vector (Promega). For *in vitro* transcription/translation the HL sequence was subcloned into *pEU3‐NII‐GLICNot* vector by ligation independent cloning 40. PIN1 localisation in protoplasts was carried out using *35S:gPIN1:GFP* 35.

Site‐directed mutagenesis reactions were performed using QuikChange Lightning Site‐Directed Mutagenesis Kit (Agilent Technologies, Santa Clara, CA, USA). Nonphosphorylatable (T227A, T248A, T286A) and phosphomimetic (T227E, T248E, T286E) PIN1 versions were generated by sequential mutagenesis reactions using the following primers: PIN1‐T248A: F: GTTCAAGAAACCCAGCGCCGCGGGGCTCTAGTTTTAATC, R: GATTAAAACTAGAGCCCCGCGGCGCTGGGTTTCTTGAAC; PIN‐T286A: F: GGTTCTAAAGGTCCTGCTCCGCGGCCTTCCAACTACG, R: CGTAGTTGGAAGGCCGCGGAGCAGGACCTTTAGAACC; PIN‐T226A: F: CGAGAAGGTCTCAAGGCTTAAGCGCTGCACCTAGACCTTCGAATC, R: GATTCGAAGGTCTAGGTGCAGCGCTTAAGCCTTGAGACCTTCTCG; PIN‐T248E: F: GAGTTCAAGAAACCCAGAGCCTAGGGGCTCTAGTTTTAATCATAC, R: GTATGATTAAAACTAGAGCCCCTAGGCTCTGGGTTTCTTGAACTC; PIN‐T286E: F: CTGTGTTTGGTTCTAAAGGTCCTGAGCCTAGGCCTTCCAACTACG, R: CGTAGTTGGAAGGCCTAGGCTCAGGACCTTTAGAACCAAACACAG; PIN‐T226E: F: CGAGAAGGTCTCAAGGCTTATCTGCAGAACCTAGACCTTCG, R: CGAAGGTCTAGGTTCTGCAGATAAGCCTTGAGACCTTCTCG. Mutant clones were verified by sequencing.

### Plant materials

*Arabidopsis thaliana* Col‐0 was used as genetic background. The T‐DNA insertion line Salk_073907 for *mpk6* was obtained from the Nottingham Arabidopsis Stock Centre. The *ProPIN1:PIN1:GFP* transgenic line was published in Ref. 41. Seeds were germinated on 0.5× Murashige and Skoog (MS) medium (Duchefa, Haarlem, The Netherlands), and plants were grown at 21–23 °C, 60–70% RH and 140 (± 20) μmol·m^−2^·s^−1^ cool white light under long day (16 h of light/8 h of dark) conditions. Transgenic Arabidopsis lines were generated using the floral dipping method 42.

### *In vitro* kinase assay

The *in vitro* mRNA synthesis was carried out using TranscriptAid T7 High Yield Transcription Kit (Thermo Scientific, Waltham, MA, USA) according to the manufacturer's instructions. Cell‐free translation was carried out by using WEPRO7240H Expression Kit (Cell Free Sciences, Yokohama, Japan). In order to activate His‐tagged MPK4 and MPK6 when included in the phosphorylation assay mix, mRNA encoding a constitutively active myc:MKK1 and myc:MKK4, respectively, were also added to the translation mixture as described 43. *In vitro*‐translated His_6_‐AtMPK4 and His_6_‐AtMPK6 proteins were purified by affinity chromatography on TALON Magnetic Beads (Clontech), *in vitro*‐translated GST‐PIN1loop and GST‐PIN1loop‐3A were purified by affinity chromatography on Glutathione Magnetic Beads (Thermo Scientific) 43.

For kinase assays, 300 and 100 ng of *in vitro*‐translated, affinity‐purified substrate and kinase were used respectively. As an activity control, 10 μg myelin basic protein was used as a generic MAPK substrate (not shown). The assays were carried out in 20 mm HEPES, pH 7.5, 100 μm ATP, 1 mm DTT, 15 mm MgCl_2_, 5 mm EGTA and 5 μCi [γ‐^32^P]ATP with bead‐bound GST‐PIN1loop or GST‐PIN1loop‐3A as substrates for 30 min at room temperature, and then stopped by the addition of Laemmli SDS buffer. Samples were fractionated by SDS/PAGE. The gel was fixed, stained with Coomassie Blue, dried and analysed by autoradiography. The kinase assay was performed three times with similar results.

### LC‐MS/MS analysis

*In vitro* phosphorylation reaction mixtures were separated on NuPAGE 4–12% Bis‐Tris gel (Thermo), bands corresponding to the GST:PIN1HL protein were excised from the gel and subjected to in‐gel digestion using side chain‐protected porcine trypsin (https://msf.ucsf.edu/protocols.html). Approximately 75% of the peptide mixtures were subjected to phosphopeptide enrichment by IMAC using Fe‐nitrilotriacetic acid 44, and the phosphopeptide fractions as well as the remaining 25% of the original samples were analysed by data‐dependent LC‐MS/MS using an Orbitrap Elite mass spectrometer. HCD and CID spectra of the three most abundant multiply charged precursor ions were acquired in each cycle (MS and HCD spectra were acquired in the Orbitrap, CID spectra in the linear ion trap; MS/MS threshold: 10 000, dynamic exclusion: 15 s). Peak lists generated from the MS/MS data by the proteome discoverer software (v. 1.4.1.14; Thermo Fisher Scientific) were searched against the Swissprot database (downloaded 12/14/2015, 550116 target sequences) using the ProteinProspector search engine (v.5.18.0.). Search parameters: enzyme – trypsin with maximum 1 missed cleavage per peptide; fixed modification – carbamidomethyl (Cys); variable modifications – acetylation (protein N terminus), oxidation (Met), pyroglutamic acid formation (peptide N‐terminal Gln) allowing maximum two variable modifications per peptide; mass accuracy – 5 p.p.m. for precursor ions and 10 p.p.m. or 0.6 Da for fragment ions (for HCD or CID data, respectively; all *m*/*z* values defined as monoisotopic). Subsequently, another search was conducted on the subset of confidently identified proteins using the same search parameters except that a maximum of two missed cleavage sites per peptide were allowed, and phosphorylation on Ser/Thr/Tyr was also set as variable modification allowing a maximum of three variable modifications per peptide. For all searches the following acceptance criteria were applied: score > 22 and 15, and *E*‐value < 0.01 and 0.05 for protein and peptide identifications respectively. For phosphopeptide site assignments, SLIP threshold 45 was set to 6. All phosphopeptide identifications were inspected manually.

### Protoplast transient expression

Protoplasts were prepared from an Arabidopsis wild‐type Col‐0 root suspension culture and transiently transformed with 5 μg of each plasmid constructs as described 46. Induction of MKK7 expression from *pER8:MKK7* was achieved by treatment with β‐estradiol for 4 h. flg22 treatment was carried by the application of 4 μm custom‐synthesised flg22 peptide for 12 h. RFP‐fused organelle markers are described in Ref. 47.

### Immunofluorescence analysis and microscopy

Samples were fixed and processed as described in Ref. 48. PIN1 was detected in permeabilised seedlings incubated with an affinity‐purified mouse anti‐PIN1 monoclonal antibody (1 : 100) and monoclonal secondary antibody (Alexa 488 goat anti‐mouse at 1 : 1000 dilution), using a Zeiss LSM 5 DUO scanning microscope (Carl Zeiss, Oberkochen, Germany). Fluorescent‐labelled anti‐PIN1 antibody and DAPI fluorescence were monitored using multitracking in frame mode. Fluorescent‐labelled anti‐PIN1 antibody was excited using the 488 nm laser line in conjunction with a 505–530 band‐pass filter. DAPI was excited with the 405 nm laser line and collected using a 420–480 nm band‐pass filter. The experiment was carried out four times with similar results. Minimally 20 roots were imaged for each genotype, the images shown are representative.

Confocal laser scanning microscopy analysis of PIN1:GFP samples was performed using a Leica TCS SP8 confocal microscope (Leica Microsystems, Wetzlar, Germany) equipped with a HC PL APO 63.0 × 1.4 OIL CS2 objective, and a GaAsP detector. Excitation/emission wavelengths of GFP and organelle‐specific RFP markers were 488/505–550 and 561/565–620 nm respectively.

### Immunoblotting

For protein gel blotting, equal protein amounts from plant extracts were separated by SDS/PAGE, transferred to polyvinylidene difluoride membranes (Millipore, Billerica, MA, USA), and probed either with horseradish peroxidase‐conjugated anti‐myc monoclonal antibody (Roche, Penzberg, Germany) or anti‐HA (Roche) and anti‐GFP (GenScript, Piscataway, NJ, USA) primary antibodies, detected by HRP‐conjucated secondary antibody (GenScript). The membranes were visualised with enhanced chemiluminescence substrate (Thermo Fisher Scientific) and exposed on CL‐Xposure film (Thermo Fisher Scientific).

## Results

### Long‐HL PINs retain highly conserved MAPK phosphorylation site patterns

**Figure 1:**
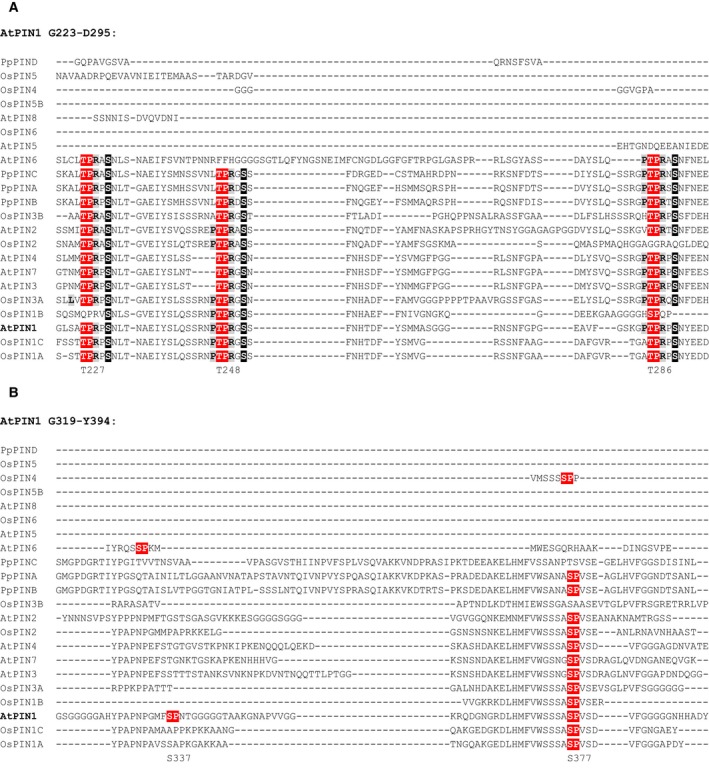
Conservation of MAPK phosphosites in PIN proteins in land plants and their co‐occurrence with PID phosphosites. Arabidopsis, rice and *Physcomitrella patens* members of the UNIPROT PIN auxin efflux protein family were aligned using the MUSCLE algorithm. The regions shown correspond to G223‐D295 (A) and G319‐Y394 (B) in the hydrophilic loop of PIN1. MAPK and PID phosphorylation sites and preferred MAPK phosphorylation flanking residues are highlighted in red, black and grey backgrounds respectively. Positions of potential MAPK sites in the full‐length PIN1 protein are indicated below. Full‐length alignment is provided in Fig. S1.

*In silico* analysis of Arabidopsis PIN protein sequences by using the Eukaryotic Linear Motif Resource (ELM) 49 identified several putative MAPK phosphorylation sites. Phylogenetic analysis revealed a strikingly high degree of conservation of three sites, suggesting a profound regulatory function. These residues correspond to T227, T248 and T286 in Arabidopsis PIN1 and are conserved in all PIN family members with extended HL sequence (Fig. 1A). Moreover, they are perfectly conserved in representative sequences from the monocotyledonous (rice) and moss (*Physcomitrella*) lineages (Fig. 1A), implying their conservation throughout land plants. Remarkably, certain amino acids corresponding to the L/P‐P/X‐S‐P‐R/K MPK6 phosphorylation preference flanking the phosphorylation site according to Ref. 50, are also present and similarly well conserved around these sites. Two of these sites, T286 and T227 were also identified as phosphorylated *in planta*, in two quantitative phosphoproteomics studies addressing stress responses 51, 52, highlighting their functional relevance. Further supportive evidence that PIN1 is a MAPK target is the presence of a MEF2A‐type MAPK docking site 53 at positions 197–206, although the first basic amino acid of this motif is missing in some PINs. In addition, it also contains a highly conserved RKLI motif at positions 471–474, which resembles the canonical MAPK docking site 54, although it lacks the spacer residue(s).

Interestingly, the three PID phosphorylation sites (S231, S252 and S290) are conserved to the same degree and are consistently located in the close proximity (i.e. four residues downstream) of the conserved putative MAPK sites. As AGC kinases do not require an upstream TP motif 49, these sites may be genuine MAPK phosphorylation target sites.

There are two additional MAPK phosphorylation sites further downstream in PIN1 HL (S337 and S377), which in contrast do not possess the flanking preference motifs and PID phosphorylation site. S337, however, was found to be a MPK6 target 38, but it is poorly conserved in the land plant PIN family (Fig. 1B). For full‐length alignment see Fig. S1.

### PIN1 is phosphorylated at T227, T248 and T286 by MPK6 and MPK4

**Figure 2:**
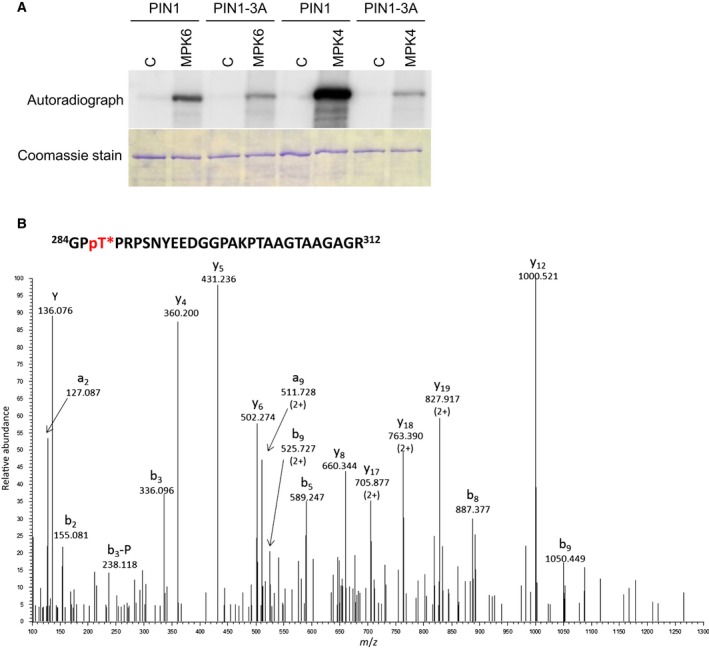
PIN1 is phosphorylated on T227, T248 and T286 by MAP kinases. (A) Kinase assay with *in vitro‐*translated, affinity‐purified wild‐type GST‐PIN1‐HL (PIN1) and T227A, T248A, T286A mutant GST‐PIN1‐HL (PIN1‐3A) variants. C: control, MPK6/MPK4: PIN1 variants incubated with *in vitro‐*translated, affinity‐purified, activated MPK6/MPK4 respectively. (B) HCD spectrum of *m*/*z*: 709.0780 (4+), representing of a phosphorylated peptide derived from MPK6‐phosphorylated PIN1 hydrophilic loop. The sequence of the precursor peptide is indicated above. Asterisk indicates phosphorylated threonine. Site of phosphorylation is Thr‐286 as proven by the phosphorylated b_3_ fragment ion. Peptide fragments are labelled according to the nomenclature by Ref. 72. ‐P stands for the 98‐Da neutral loss of phosphoric acid characteristic to Ser/Thr phosphorylation.

Wide‐ranging conservation of MAPK phosphorylation site patterns and their co‐occurrence with the PID sites along with proven *in planta* phosphorylation suggest functional importance of T227, T248 and T286, therefore we tested their MAPK‐mediated phosphorylation. To this end, *in vitro* kinase assays were performed first (Fig. 2A). As shown by radiolabelled phosphate incorporation, the *in vitro*‐translated and purified hydrophilic loop of the wild‐type PIN1 was phosphorylated by MPK6. In contrast, substitution of the three MAPK phosphorylation residues with nonphosphorylatable alanines (T227A/T248A/T286A) resulted in a marked diminution of phosphorylation. These results indicate that T227, T248 and T286 are MAPK phosphorylation sites on PIN1. To directly demonstrate the MPK6‐mediated phosphorylation of these residues we performed LC‐MS/MS analyses of the tryptic digests of the MPK6‐treated and control GST‐PIN1 HL. Phosphorylation of T227, T248, T286 and the previously reported S337 38 was verified in the kinase‐treated samples by the identification of five phosphopeptides (one doubly phosphorylated) (Fig. 2B, Fig. S2). The same results were obtained both with and without phosphopeptide enrichment, in two repeats.

The conservation of the MAPK phosphorylation sites corresponding to T227, T248 and T286 of PIN1 down to the *Physcomitrella* long‐HL PIN sequences implies a very early formation of the MAPK‐PIN regulatory link during land plant evolution. Separation of the group A and B MAPK clades most probably took place after the separation of the flowering plant lineage from more ancient lineages such as mosses and lycophytes, possibly as an adaptation to the formation of complex body architectures 55. This raises the possibility that PIN1 was retained as a substrate following the emergence of novel MAPKs through gene duplications, thus also raising the possibility that PIN1 is phosphorylated by group B MAPKs as well. To test this hypothesis we investigated the phosphorylation of PIN1 by MPK4, the best‐characterised member of plant group B MAPKs. Similarly to MPK6, MPK4 phosphorylates PIN1 and this phosphorylation is strongly reduced when T227, T248 and T286 are rendered nonphosphorylatable (Fig. 2A).

### Inducible expression of MKK7 and flagellin treatment modulate plasma membrane localisation of PIN1 in roots

**Figure 3:**
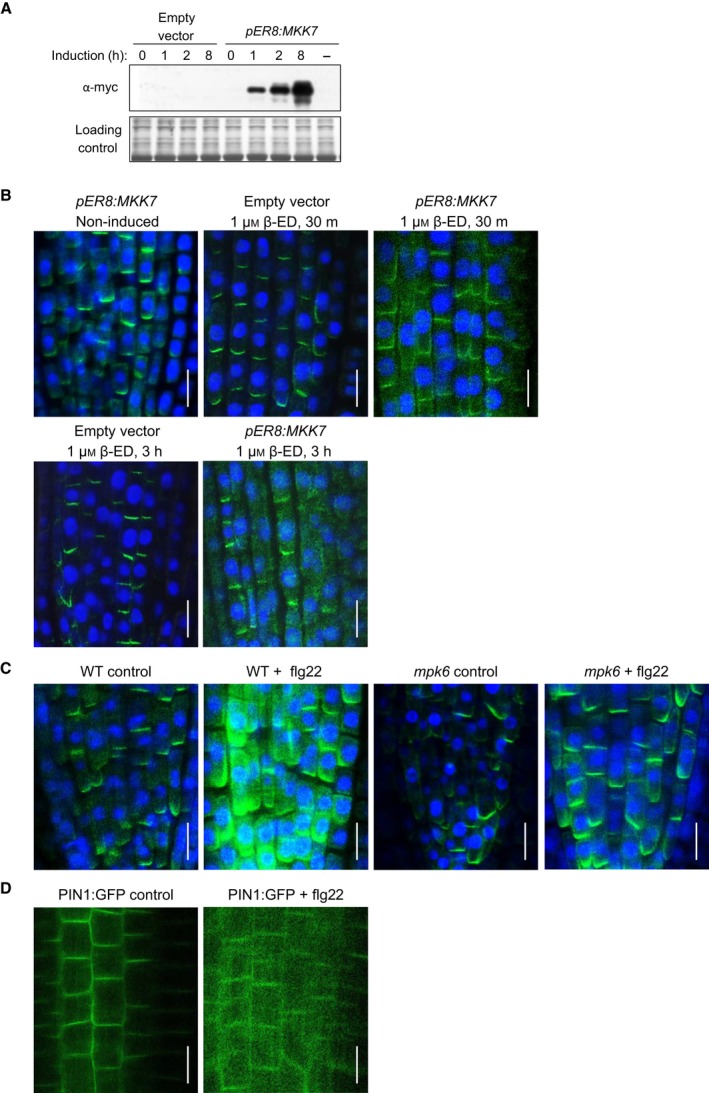
MKK7 overexpression and flagellin treatment lead to PIN1 delocalisation in roots. (A) Detection of transgenic MKK7 expression. Expression of inducible myc:MKK7 (*pER8:MKK7*) protein was detected by immunoblotting, using an anti‐myc antibody. Duration of induction by 1 μm β‐estradiol is indicated in hours. Equal protein loading was visualised by Ponceau staining of the blotted membrane. (B) Immunolocalisation of PIN1 in roots of empty vector and *pER8:MKK7* seedlings. Induction conditions are indicated. (C) Immunolocalisation of PIN1 in roots of untreated or flg22‐treated (10 μm, 24 h) wild‐type or *mpk6* mutant seedlings. (D) PIN1 localisation in roots of control and flg22‐treated *ProPIN1:PIN1:GFP* transgenic seedlings. (B–D) Scale bar: 10 μm.

Moderate overexpression of MKK7 in the *bud1* mutant inhibits PAT and leads to developmental anomalies and the MKK7‐MPK6 module has been reported to modulate PIN1 polarity in the xylem parenchyma cells of the basal inflorescence stems 38. We used transgenic lines that express wild‐type *MKK7*, which was shown to activate downstream MAPKs 38, under the control of a β‐estradiol‐inducible promoter system (pER8:MKK7) (Fig. 3A). MKKs normally require activation through the T/SXXXXXT/S phosphorylation site 55. However, MKK7 has an Asp residue at position 3 (SLDYCNS), which corresponds to the position of the phosphorylation site of animal MKKs. Such plant MKKs are autoactive 56, 57. MAPK activation in induced pER8:MKK7 seedlings will be shown elsewhere 58. We tested if MKK7 overexpression also alters PIN1 localisation in root tips of young seedlings, a system that is well‐characterised for PIN1 localisation. In the root tip, PIN1 is localised to the stele and cortex cells as well as the initials. In these cells, PIN1 is almost always localised basally with the exception of a few cortex cells where it may also localise apically 41, 59.

As a negative control we used empty vector‐transformed seedlings, where PIN1 localisation was normal even when treated with β‐estradiol (Fig. 3B). Seedlings carrying the inducible *MKK7* transgene not treated with β‐estradiol also presented a normal PIN1 localisation pattern (Fig. 3B). However, when such seedlings were treated with β‐estradiol to induce the expression of MKK7, PIN1 PM localisation was partially lost and PIN1 accumulated in intracellular patches (Fig. 3B). This rapid delocalisation of PIN1 in root cells suggests a direct post‐translational regulation by MAPK.

flg22, a peptide derived from the bacterial elicitor, flagellin, is a well‐characterised activator of MPK6 60 and we also tested if flg22‐triggered MPK6 activation affects the cellular localisation of PIN1. Basal PM localisation was observed in untreated control seedlings, whereas flg22 treatment led to a similar mislocalisation as observed in MKK7‐overexpressing samples, that is, partial loss of PM localisation with the appearance of an intracellular, patchy PIN1 fraction (Fig. 3C,D). Similar results were obtained by two independent experimental approaches, that is, immunolocalisation of endogenous PIN1 (Fig. 3C) or by using *ProPIN1:PIN1:GFP* transgenic seedlings (Fig. 3D). In contrast, in *mpk6* T‐DNA insertion mutant seedlings the flg‐triggered delocalisation was abolished (Fig. 3C), indicating that MPK6‐mediated phosphorylation regulates PIN1 localisation.

### Phosphorylation status of T227, T248 and T286 influences PIN1 localisation in protoplasts

**Figure 4:**
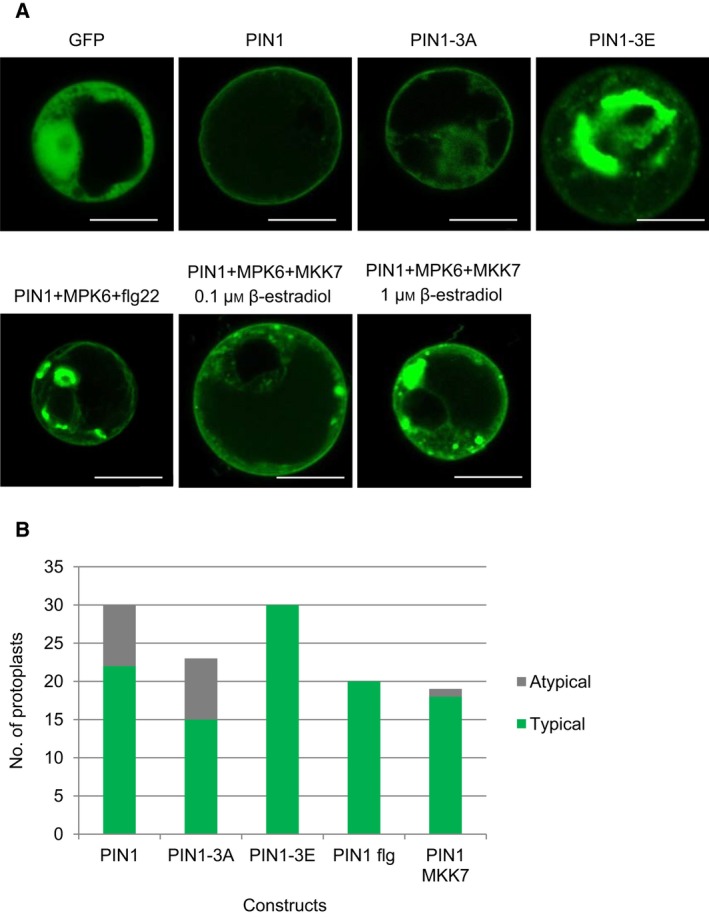
MAPK phosphorylation status at T227, T248 and T286 influences intracellular trafficking of PIN1 in root‐derived protoplasts. (A) Protoplasts were transformed with the indicated constructs and subjected to the indicated treatments. All experiments were carried out minimally three times. Scale bar: 10 μm. (B) Quantification of protoplasts with typically localised PIN1:GFP variants as shown in the representative images. Typically WT PIN1 is exclusively localised to the plasma membrane (PM). Protoplasts with any internal GFP fractions, including patches and aggregates are categorised as atypical for PIN1. PIN1‐3A typically localised internally besides the PM, without patches or aggregates. PIN1‐3A samples where patches or aggregates were formed are categorised as atypical. Protoplasts transfected with PIN‐3E, PIN1/MPK6 + flg22 and PIN1/MPK6/MKK7 typically formed internal aggregates. In a total of 69 such protoplasts analysed only one was found where the PM‐localised GFP signal was exclusively maintained.

In protoplasts, PIN1 is plasma membrane (PM) localised even when it is overexpressed, and therefore this experimental system was previously utilised to study the regulation of PIN1 PM localisation 32, 35. We used the same experimental system of transient transformation of the *35S:PIN1:GFP* fusion construct into protoplasts to study the effect of MAPK phosphorylation at T227, T248 and T286 on intracellular localisation of PIN1. The expression of cotransformed PIN1:GFP variants, MPK6:HA and myc:MKK7 were verified by western blots (Fig. S3A). Representative images taken from 20 to 30 cells in at least three independent experiments are shown (Fig. 4, Fig. S3). In protoplasts isolated from a root‐derived cell culture PIN:GFP localised to the plasma membrane (Fig. 4). When coexpressed with MPK6 and either treated with flg22 or coexpressed with induced MKK7, PIN1 accumulated intracellularly, forming aggregates, similarly to what was observed in root cells (Fig. 4, Fig. S3B), implying that root‐derived protoplasts offer a suitable model to investigate the effect of altered phosphorylatability.

Expression of a phosphomimetic mutant version of the sites in question on PIN1:GFP (T227E/T248E/T286E) resulted in alterations of PIN1:GFP subcellular localisation similar to that observed in response to MPK6 activation, but in an exaggerated form: aggregates of extremely large size were commonly formed (Fig. 4, Fig. S3C,D). The nonphosphorylatable PIN1 mutant (T227A/T248A/T286A) also appeared in intracellular fractions, but it did not form any patches or aggregates (Fig. 4, Fig. S3E,F).

In order to associate intracellular PIN1 fractions with specific organelles PIN1:GFP variants were cotransformed with RFP‐fused organelle markers 47. Overphosphorylated PIN1 forms (either phosphomimetic or MKK7 coexpressed) do not associate unambiguously with either the ER or the Golgi marker (Fig. S3B–D). However, the large aggregates associate with both markers. Similarly, there is only partial overlap between the nonphosphorylatable PIN1 mutant and the Golgi marker (Fig. S3F). Remarkably, the ER marker associates well with this mutant version (Fig. S3E). Taken together these results imply that PIN1 phosphorylation by MKK7‐MPK6 at T227, T248 and T286 influences its intracellular accumulation, presumably by interfering with trafficking mechanisms.

## Discussion

Unlike animal development – where organs are formed early during embryogenesis largely independently of environmental factors – organogenesis and organ development in sessile plants continues throughout the organism's life and flexibly respond to changing environmental conditions. Key to this continuous adaptation is the cross‐talk between environmental and developmental signalling mechanisms. Taken together here we provide biochemical evidence for the existence of a possibly ancient regulatory link between PAT, a key mechanism of adaptive growth regulation and environmentally activated MAPK signalling, by demonstrating MAPK‐mediated phosphorylation of highly conserved residues of PIN1, which play a role in its intracellular localisation.

Phosphorylation status is already well known as an important regulatory factor of PIN subcellular targeting. Identified molecular components regulating the polarity of PIN proteins are the protein kinase PID 30, 31 and related kinases of the AGC family WAG1 and WAG2 32, as well as PP2A phosphatases 35. PID gain‐of‐function results in an opposite basal‐to‐apical PIN polarity shift leading to auxin depletion from the root meristem, ultimately leading to its collapse 33. Our results reveal that T227, T248, T286, in close proximity of the PID phosphosites, are phosphorylation sites of MPK6 and MPK4, two environmental stress‐activated MAP kinases. Accordingly, T286 and T227 were identified as phosphorylated *in vivo* in response to stress treatments in two mass spectrometry‐based quantitative phosphoproteomics studies, indicating possible MAPK‐mediated phosphorylation 51, 52. The three novel sites sufficiently meet described features of MAPK phosphorylation site patterns: clustering of MAPK phosphorylation sites on substrate proteins is relatively common 61 and the preferred flanking residues 50 are present to a large extent and there are potential docking motifs.

Unlike PID, MKK7 overexpression does not lead to a shift in PIN1 polarisation in roots, suggesting that such phosphorylated PIN forms enter a different protein trafficking route from those forms phosphorylated by AGC3 kinases. Similarly to the PID phosphorylation sites, the identified MAPK phosphorylation sites are perfectly conserved in land plants in PINs possessing long‐HL sequences. Remarkably, nonphosphorylatable PIN1 associates with the ER marker in protoplasts, similarly to the fate of short‐HL PINs, lacking the conserved MAPK phosphorylation sites. Phosphomimetic PIN1 on the other hand is entrapped into intracellular formations. Considering the dynamic nature of phosphorylation, nonphosphorylatable and phosphomimetic mutants represent states where the phosphorylation equilibrium is tilted to either extreme. Accordingly, localisation of these mutants suggests that a balanced MAPK phosphorylation is required for proper PIN1 trafficking to establish its plasma membrane localisation. It is noteworthy that other phosphorylation events studied so far affected PIN polarity within the PM but did not alter the PM targeting of PIN. In this scenario MAPK phosphorylation may regulate access to particular trafficking mechanisms, which bring about or prevent plasma membrane localisation, while PID phosphorylation on adjacent sites regulates apical versus basal polarity determination.

The co‐occurrence of T227/T248/T286 sites with the conserved PID phosphorylation sites at adjacent positions suggests a regulatory coevolution. Interestingly, the + 2 R residues downstream of the three MAPK phosphosites are part of both the MPK3/6 preference motif and the preference motif of the Protein Kinase A (PKA) group. Conservation of ‘double preference’ residues may be plausibly interpreted as indirect evidence of the coevolution of two phosphorylation sites through a motif merger. Thus, functionality of MAPK‐mediated phosphorylation may be at least as complex as in the case of PID, requiring further studies to dissect details of this novel regulatory mechanism.

It was recently reported that PIN1 is also phosphorylated by MPK6 at S337 located C‐terminally at a distinct region and the phosphorylation status of this residue apparently participates in the regulation of PIN1 polarity in a specific developmental setting of xylem parenchyma cells in 35‐day‐old basal inflorescence stems 38. However, unlike T227/T248/T286, S337 is poorly conserved in land plant PIN family, suggesting that this phosphorylation event might be involved in a specific fine‐tuning of PIN1 function.

Polar auxin transport and the resulting local auxin maxima sites are important in establishing developmental patterns in plants. PINs determine the direction of PAT through their asymmetric subcellular localisation and thus signalling pathways regulating PIN localisation can modulate developmental programs in response to triggering stimuli 62, 63. In this framework it is conceivable that environmentally activated MAPK pathways participate in modulating PIN trafficking in response to environmental cues. MAPK activation by flagellin 8, 64 or salt 10, 65 sensing are well‐described. Internalisation of PIN2 in root halotropic response was also described 66, 67. Our results thus raise the possibility that salt‐induced intracellular fractions of PIN2 are a consequence of MAPK‐mediated phosphorylation. Similarly, here we show flg22‐triggered delocalisation of PIN1, and by using *mpk6* mutant we also demonstrate that this is an MPK6‐mediated process. Considering that flg22 activates further MAPKs besides MPK6, including MPK4, this result was rather unexpected. A plausible explanation is differential accessibility of PIN1 through kinase localisation (regulation through compartmentalisation). Expression and cellular localisation of both MPK6 and MPK4 in root cells have been studied in detail. While MPK6 is localised to the trans‐Golgi network and the plasma membrane 68, strongly supporting its role in modulating PIN1 trafficking, MPK4 protein is predominantly localised to the nucleus, with cytoplasmic fractions being also detectable both in mesophyll 69 and root 22, 70 cells. This makes PIN1 phosphorylation by MPK4 under standard conditions less feasible. Nonetheless, as protein localisation is highly dynamic, for example, in dividing root cells MPK4 becomes localised to cell plates 22, MPK4‐mediated PIN1 phosphorylation *in planta* under specific conditions cannot be ruled out entirely. A flagellin‐induced delocalisation of PIN1 is also in line with the severe retardation of root growth in response to flagellin treatment 71.

The MAPK pathways are central signal transduction pathways in all eukaryotes. In both mammals and flowering plants they regulate a plethora of processes. Understanding the mechanisms of such complexity requires a detailed dissection of MAPK networks, yet our knowledge on MAPK substrate phosphorylation in plants is limited. Our results provide evidence for the co‐option of MAPKs together with AGC kinases at an early stage of land plant evolution to regulate PM‐localised PIN targeting, in line with the challenges of terrestrial lifestyle. Environmentally activated MAPK pathways constitute a novel regulatory mechanism of PIN, which may play a role in the environmental plasticity of plant development.

## Supporting information


**Fig. S1.** Sequence alignment of Arabidopsis, rice and *Physcomitrella patens* members of the UNIPROT PIN auxin efflux protein family.
**Fig. S2.** Spectra for representative identified phosphopeptides derived from PIN1 hydrophilic loop.
**Fig. S3.** MAPK phosphorylation status at T227, T248 and T286 influences intracellular trafficking of PIN1.
