# Supplementary material for: Coevolving MAPK and PID phosphosites indicate an ancient environmental control of PIN auxin transporters in land plants
Source: FEBS Lett. 2017 Dec 25;592(1):89–102. doi: 10.1002/1873-3468.12929 (PMC5814726; doi:10.1002/1873-3468.12929)
Supplement: Supplementary file 1 — Fig. S1. Sequence alignment of Arabidopsis, rice and Physcomitrella patens members of the UNIPROT PIN auxin efflux protein family. Fig. S2. Spectra for representative identified phosphopeptides derived from PIN1 hydrophilic loop. Fig. S3. MAPK phosphorylation status at T227, T248 and T286 influences intracellular trafficking of PIN1. [file FEB2-592-89-s001.pdf]

Supporting Information

Dory *et al.* – Co-evolving MAPK and PID Phosphosites Indicate an Ancient Environmental Control of PIN Auxin Transporters in Land Plants

```
PpPIND          MLTGGQFYDVLCAVVPLYVALFLGYGSLKWWGVVTPEQSAGISRFNALIAMPPLIFEIIA
OsPIN5          MITGSEVYQVVEAMAPLYTAAALGYGSVRWLKAFSNEQCAGINHFVALYAVPVLIFDMVS
OsPIN4          MIGWGDVYKVVGAMAPLYFALGLGYGSVRWWRFFTAEQCAAINTMVVYFSMPFFTFDFVV
OsPIN5B         MIGWGDVYKVVAAMAPLYFALGLGYGSVRWWRLFTADQCDAVNRLVACFAVPFFAFDFAA
AtPIN8          MINCGDVYKVIEAMVPLYVALILGYGSVKWWHIFTRDQCDAINRLVCYFTLPLFTIEFTA
OsPIN6          MIGWGDVYKVVAATVPLYFALFLGYGSVRWWRIFTREQCDAVNRLVAFFALPFFTFEFTL
AtPIN5          MISWLDIYHVVSATVPLYVSMTLGFLSARHLKLFSPEQCAGINKFVAKFSIPLLSFQIIS
AtPIN6          MITGNEFYTVMCAMAPLYFAMFVAYGSVKWCKIFTPAQCSGINRFVSVFAVPVLSFHFIS
PpPINC          MITGHDMYNVLSAMVPLYVAMMLAYASVKWWGILTPQQCDGINRFVSIFAVPLLSFQFVS
PpPINA          MINGHDIYNVLSAMVPLYVAMMLAYGSVKWWGILTPQQCGGINRFVSIFAVPLLSFQFIS
PpPINB          MINGHDIYNVLSAMVPLYVAMMLAYGSVKWWGILTPQQCGGINRFVSIFAVPLLSFQFIS
OsPIN3B         MISWHELYMVLSAVVPLYVAMMVAYGSVRWWGVLTPEQCSGINRFVAVIAVPLLSFHFIS
AtPIN2          MITGKDMYDVLAAMVPLYVAMILAYGSVRWWGIFTPDQCSGINRFVAVFAVPLLSFHFIS
OsPIN2          MITGRDIYDVLAAIVPLYVAMFLAYGSVRWWGIFTPDQCSGINRFVAVFAVPLLSFHFIS
AtPIN4          MITWHDLYTVLTAVVPLYVAMILAYGSVQWWKIFSPDQCSGINRFVAIFAVPLLSFHFIS
AtPIN7          MITWHDLYTVLTAVIPLYVAMILAYGSVRWWKIFSPDQCSGINRFVAIFAVPLLSFHFIS
AtPIN3          MISWHDLYTVLTAVIPLYVAMILAYGSVRWWKIFSPDQCSGINRFVAIFAVPLLSFHFIS
OsPIN3A         MISGHDFYTVMAAVVPLYVAMFLAYGSVRWWGIFTPDQCSGINRFVAIFAVPLLSFHFIS
OsPIN1B         MITVVDLYHVLTAVVPLYVAMTLAYASVRWWRIFSPDQCSGINRFVALFAVPLLSFHFIS
AtPIN1          MITAADFYHVMTAMVPLYVAMILAYGSVKWWKIFTPDQCSGINRFVALFAVPLLSFHFIA
OsPIN1C         MITGADFYHVMTAMVPLYVAMILAYGSVKWWRIFTPDQCSGINRFVALFAVPLLSFHFIS
OsPIN1A         MITAADFYHVMTAMVPLYVAMILAYGSVKWWRIFTPDQCSGINRFVALFAVPLLSFHFIS
                *:   :.* *: *  *** :  :.: * ..   .:  *. .:. :    ::* : : :
                                                  T35

PpPIND          FNNPYTMNNRLIAAYCLSNGIVLVGLCAWV-----WCT-------KCG----N--LD-WV
OsPIN5          TNNVYKMNGRLIAADTLQKAVLLLGLMAWAL----WERSRARGAGAKAKAAVSSPLQ-WV
OsPIN4          RTDPFAMNYRVIAADAVSKAIAIAAMAAWAR------T-------RCGCAAAKAGAQSWS
OsPIN5B         RIDPFALSYRVLAADALSKLAVALALAACAAAASTR-CCGSGGGKRGG----GGGFS-WC
AtPIN8          HVDPFNMNYRFIAADVLSKVIIVTVLALWAK----Y-S-------NKG----S--YC-WS
OsPIN6          HTDPFQVNYRAVAADVISKAVIVAVIGAWAR----F-M-------SKG----GCAVS-WS
AtPIN5          ENNPFKMSPKLILSDILQKFLVVVVLAMVLR----FWH-------PTGGRGGK--LG-WV
AtPIN6          QNNPYKMDTMFILADTLSKIFVFVLLSLWAV----F-F-------KAG----G--LD-WL
PpPINC          GNNPYEMNFRFIAADAVSKVFVLSCLGLWVR----F-S-------KRG----S--LE-WV
PpPINA          GNNPYAMNFKFIAADAVSKVLVLLCLGLWAR----Y-A-------KRG----S--LE-WM
PpPINB          GNNPYAMNFRFIAADAVSKVFVLLCLGLWAR----Y-S-------KRG----S--LE-WM
OsPIN3B         SSDPYAMNLRFVAADTLQKVLVLAALAAWSRFPARF-V-------PPA----WPPLD-CS
AtPIN2          SNDPYAMNYHFLAADSLQKVVILAALFLWQA----F-S-------RRG----S--LE-WM
OsPIN2          TNDPYSMNYRFLAADSLQKLVILAALAVWHNLLSRY-R-------RNGGAAAS--LD-WT
AtPIN4          TNDPYAMNFRFVAADTLQKIIMLVLLALWAN----L-T-------KNG----S--LE-WM
AtPIN7          SNNPYAMNLRFIAADTLQKLIMLTLLIIWAN----F-T-------RSG----S--LE-WS
AtPIN3          TNNPYAMNLRFIAADTLQKIIMLSLLVLWAN----F-T-------RSG----S--LE-WS
OsPIN3A         TNDPYAMNLRFLAADTLQKLLVLAGLAAWSR----LPS-------RTG----APRLD-WS
OsPIN1B         TNNPFAMNLRFLAADTLQKLIVLALLALWCR----L-S-------ARG----S--LD-WL
AtPIN1          ANNPYAMNLRFLAADSLQKVIVLSLLFLWCK----L-S-------RNG----S--LD-WT
OsPIN1C         TNNPYTMNLRFIAADTLQKLIVLALLTLWSH----L-S-------RRG----S--LE-WT
OsPIN1A         TNNPYTMNLRFIAADTLQKLMVLAMLTAWSH----L-S-------RRG----S--LE-WT
                  : : :.   : :  :.:      :                     .

PpPIND          ITLFQLSVMPNTIIVGIPVLSPLYS------VTESGIAAIFIGQVLWLFPTLFLYELKEV
OsPIN5          ITCFSVASLPNTIIMGVPLLNGMYGPV----SKDLMKQIVVMQFCIWYNVIIFLYEYMAA
```

```
OsPIN4          ITGFSLAALNNTLVVGVPLLDAMYGRW----AQDLVVQIAVVQSMVWFPLLLMAFELRKA
OsPIN5B         ITGFSLATLNNTLVVGVPLLDAMYGKW----ARDLIVQISVVQTIVYFPLLLLAFEVRRA
AtPIN8          ITSFSLCTLTNSLVVGVPLAKAMYGQQ----AVDLVVQSSVFQAIVWLTLLLFVLEFRKA
OsPIN6          ITSFSLSTLTNSLVVGVPMARAMYGEW----AQQLVVQLSVFQAIVWLTLLLFVLEVRKA
AtPIN5          ITGLSISVLPNTLILGMPILSAIYGDE----AASILEQIVVLQSLIWYTILLFLFELNAA
AtPIN6          ITLFSIATLPNTLVMGIPLLQAMYGDY----TQTLMVQLVVLQCIIWYTLLLFLFELRAA
PpPINC          ITLFMLTTIPNTLVIGTPLLAAMYGSK----PGQLTVQAVVLQCIIWYTLLLVMYEYRAA
PpPINA          ITLFVLITIPNTLVMGTPLLAAMYGAG----PGDLTVQAVVLQCIIWYTLLLVMYEYRAA
PpPINB          ITLFVLITIPNTLVMGTPLLAAMYGPG----PGDLTIQAVVLQCIIWYTLLLLMYEYRAA
OsPIN3B         ITLFSVSTLPNTLVMGIPLLVSMYGPY----SGDLMVQIVVLQSIVWYTLLLFLFEFRAA
AtPIN2          ITLFSLSTLPNTLVMGIPLLRAMYGDF----SGNLMVQIVVLQSIIWYTLMLFLFEFRGA
OsPIN2          ITLFSLSTLPNTLVMGIPLLRAMYGDF----SGSLMVQIVVLQSVIWYTLMLFLFEYRGA
AtPIN4          ITIFSLSTLPNTLVMGIPLLIAMYGTY----AGSLMVQVVVLQCIIWYTLLLFLFEYRGA
AtPIN7          ITIFSLSTLPNTLVMGIPLLIAMYGEY----SGSLMVQIVVLQCIIWYTLLLFLFEYRGA
AtPIN3          ITIFSLSTLPNTLVMGIPLLIAMYGEY----SGSLMVQIVVLQCIIWYTLLLFLFEFRGA
OsPIN3A         ITLFSLSTLPNTLVMGIPLLIAMYGPY----SGSLMVQIVVLQCIIWYTLMLFLFEFRAA
OsPIN1B         ITLFSLSTLPNTLVMGIPLLKGMYAAADVDSGSLMVQIVVLQCIIWYTLMLFLFEYRGA
AtPIN1          ITLFSLSTLPNTLVMGIPLLKGMYGNF----SGDLMVQIVVLQCIIWYTLMLFLFEYRGA
OsPIN1C         ITLFSLSTLPNTLVMGIPLLKGMYGEF----SGSLMVQIVVLQCIIWYTLMLFMFEYRGA
OsPIN1A         ITLFSLSTLPNTLVMGIPLLKGMYGEF----SGSLMVQIVVLQCIIWYTLMLFMFEYRGA
                ** :  :  : *::::* *:   :*.               ..   ::    :.  *   .

PpPIND          RKM---------------------------------------------------------
OsPIN5          RRSASAPPPASSE--------GSAKISP------SSPVKAAAAAADT-------------
OsPIN4          WVV---------------------------------------------------------
OsPIN5B         TTAAAAPPPPPTG-----------------------------------------------
AtPIN8          GF----------------------------------------------------------
OsPIN6          AIGMYVDGAEAAA-----------------------------------------------
AtPIN5          RAL-----PSSGA-------------SL--------------------------------
AtPIN6          RLLIRAEFPGQAAGSIAKIQVDDDVISL---DG-MDPLRTETETDVNGRIRLRIRRSVSS
PpPINC          RILIMHRFPENAA-SIVSFKVESDVMSL---DG-PDPVLTEAEFRNDGKLHVRVRRSVSS
PpPINA          KILIMQQFPENAA-SIVSFKVDSDVMSL---DG-REPVLTEAEIGDDGKLHVKVRRSVSS
PpPINB          KILIMQQFPENAG-SIVSFKVDSDVMSL---DG-REPVLTEAEIGDDGKLHVKVRRSVSS
OsPIN3B         RVLIAAQFPDTAA-SIAAVHVDPDVVSL---EGSQA--EAHAEVAPDGRLRMVVCRSSVS
AtPIN2          KLLISEQFPETAG-SITSFRVDSDVISL---NG-REPLQTDAEIGDDGKLHVVVRRSSAA
OsPIN2          KALISEQFPPDVGASIASFRVDSDVVSL---NG-REALQADAEVGRDGRVHVVIRRSASA
AtPIN4          KLLIMEQFPETGA-SIVSFKVESDVVSL---DG-HDFLETDAEIGNDGKLHVTVRKSNAS
AtPIN7          KILIMEQFPETGA-SIVSFKVESDVVSL---DG-HDFLETDAQIGDDGKLHVTVRKSNAS
AtPIN3          KMLIMEQFPETAA-SIVSFKVESDVVSL---DG-HDFLETDAEIGDDGKLHVTVRKSNAS
OsPIN3A         RMLIADQFPDTAA-SIVSLHVDPDVVSL---EGGHA--ETEAEVAADGRLHVTVRRSSVS
OsPIN1B         RLLVMEQFPDTAA-SIVSFRVDSDVVSLAGGGGGAAELQAEAEVGDDGRMRVTVRKSTSS
AtPIN1          KLLISEQFPDTAG-SIVSIHVDSDIMSL---DG-RQPLETEAEIKEDGKLHVTVRRSNAS
OsPIN1C         RILITEQFPDTAG-AIASIVVDADVVSL---DGRRDMIETEAEVKEDGKIHVTVRRSNAS
OsPIN1A         RMLITEQFPDTAA-NIASIVVDPDVVSL---DGRRDAIETETEVKEDGRIHVTVRRSNAS


PpPIND          -----------------------GQPAVGSVA----------------------------
OsPIN5          ------------------NGNAVAADRPQEVAVNIEITEMAAS---TARDGV--------
OsPIN4          -------------------------------------------------GGG--------
OsPIN5B         ------------------------------------------------------------
AtPIN8          --------------------------SSNNIS-DVQVDNI--------------------
OsPIN6          ------------------------------------------------------------
AtPIN5          ------------------------------------------------------------
AtPIN6          --------VPDSVM----SSSLCLTPRASNLS-NAEIFSVNTPNNRFFHGGGGSGTLQFY
PpPINC          R--SQGVHSANHSI----PSSKALTPRASNLS-NAEIYSMNSSVNLTPRGSS--------
PpPINA          R--SQGMHSAHHSM----PSSKALTPRPSNLT-GAEIYSMHSSVNLTPRDSS--------
PpPINB          R--SQGMHSAHHSM----PSSKALTPRPSNLT-GAEIYSMHSSVNLTPRDSS--------
OsPIN3B         ---RRSAAA-------------AATPRASNLT-GVEIYSISSSRNATPRGST--------
AtPIN2          SSMISSFNKSHGGG----LNSSMITPRASNLT-GVEIYSVQSSREPTPRASS--------
```

```
OsPIN2          STTGGGGGAARSGVSRAYGASNAMTPRASNLT-GVEIYSLQTSREPTPRASS--------
AtPIN4          ------------------RRSLMMTPRPSNLT-GAEIYSLSS----TPRGSN--------
AtPIN7          ---RRSFYG---------GGGTNMTPRPSNLT-GAEIYSLNT----TPRGSN--------
AtPIN3          RRS---------------FCGPNMTPRPSNLT-GAEIYSLST----TPRGSN--------
OsPIN3A         ------------------RRSLLVTPRPSNLT-GAEIYSLSSSRNPTPRGSN--------
OsPIN1B         RSEAACSHGTQ-------SHSQSMQPRVSNLS-GVEIYSLQSSRNPTPRGSS--------
AtPIN1          ----RSDIYSRRSQ------GLSATPRPSNLT-NAEIYSLQSSRNPTPRGSS--------
OsPIN1C         ----RSDVYSRRSM-----GFSSTTPRPSNLT-NAEIYSLQSSRNPTPRGSS--------
OsPIN1A         ----RSDIYSRRSM----GFS-STTPRPSNLT-NAEIYSLQSSRNPTPRGSS--------
                                        T227                  T248

PpPIND          -------------------------------QRNSFSVA---------------------
OsPIN5          ------------------------------------------------------------
OsPIN4          ---------------------------------------------------GGVGPA---
OsPIN5B         ------------------------------------------------------------
AtPIN8          ------------------------------------------------------------
OsPIN6          ------------------------------------------------------------
AtPIN5          ---------------------------------------------------EHTGNDQEE
AtPIN6          NGSNEIMFCNGDLGGFGFTRPGLGASPR---RLSGYASS-----DAYSLQ-----PTPRA
PpPINC          -------FDRGED--CSTMAHRDPN------RKSNFDTS-----DIYSLQ-SSRGPTPRN
PpPINA          -------FNQGEF--HSMMSQRSPH------RQSNFDTS-----DVYSLQ-SSRGPTPRS
PpPINB          -------FNQGEY--FSMMAQRSPH------RQSNFDIS-----DVYSLQ-SSRGPTPRT
OsPIN3B         -------FTLADI--------PGHQPPNSALRASSFGAA-----DLFSLHSSSRQHTPRP
AtPIN2          -------FNQTDF--YAMFNASKAPSPRHGYTNSYGGAGAGPGGDVYSLQ-SSKGVTPRT
OsPIN2          -------FNQADF--YAMFSGSKMA-------------S-----QMASPMAQHGGAGGRA
AtPIN4          -------FNHSDF--YSVMGFPGG-------RLSNFGPA-----DLYSVQ-SSRGPTPRP
AtPIN7          -------FNHSDF--YSMMGFPGG-------RLSNFGPA-----DMYSVQ-SSRGPTPRP
AtPIN3          -------FNHSDF--YNMMGFPGG-------RLSNFGPA-----DMYSVQ-SSRGPTPRP
OsPIN3A         -------FNHADF--FAMVGGGPPPPTPAAVRGSSFGAS-----ELYSLQ-SSRGPTPRQ
OsPIN1B         -------FNHAEF--FNIVGNGKQ--------------G-----DEEKGAAGGGGHSPQP
AtPIN1          -------FNHTDF--YSMMASGGG-------RNSNFGPG-----EAVF---GSKGPTPRP
OsPIN1C         -------FNHTDF--YSMVG-----------RSSNFAAG-----DAFGVR---TGATPRP
OsPIN1A         -------FNHTDF--YSMVG-----------RSSNFGAA-----DAFGVR---TGATPRP
                                                                    T286

PpPIND          ------------------------------------------------------------
OsPIN5          ------------------------------------------------------------
OsPIN4          ------------------------------------------------------------
OsPIN5B         ------------------------------------------------------------
AtPIN8          ------------------------------------------------------------
OsPIN6          ------------------------------------------------------------
AtPIN5          ANIEDEP-----------------------------------------------------
AtPIN6          SNFNELD-----------------------VNGNGTPVWMKSPAA-----------GR--
PpPINC          SNFNEENSKEVHNHRGALNVNIPRFAPPLYRNGSGGRLFMARSDLGGVGALSFEPAAH--
PpPINA          SNFNEENSKDIHTHHRGLNMNSPRFAPPLYRNGMGARMFTPRPGLGGIGVPGTDCTGHGT
PpPINB          SNFNEENSKDMHTHHRGLNLTSPRFVPPLYRNVAGGRMFMPRTGLGGLPVHGNDPTGHGS
OsPIN3B         SSFDEHA-------------------------------------------------AA--
AtPIN2          SNFDEEV------------------MKTAKKAGRGGRSMSGEL-----------------
OsPIN2          QGLDEQV---------------------TNKFASGKAADPPS------------------
AtPIN4          SNFEENN-------------------AVKYGFYNNTNSSVPAAGS---------------
AtPIN7          SNFEESCAMA---------------SSPRFGYYPGGAPGS--------------------
AtPIN3          SNFEENCAMA---------------SSPRFGYYPGGGAGS--------------------
OsPIN3A         SNFDEHSA----------------------------------------------------
OsPIN1B         ------------------------------------------------------------
AtPIN1          SNYEEDGGPA---------------KPTAAGTAAGAGRFHYQSG----------------
OsPIN1C         SNYEEDAA-----------------APNKAGSKYGQ------------------------
OsPIN1A         SNYEDDA------------------SKPKYPLPASNAAPMAGH-----------------
```

```
PpPIND          ------------------------------------------------------------
OsPIN5          ------------------------------------------------------------
OsPIN4          ------------------------------------------------------------
OsPIN5B         ------------------------------------------------------------
AtPIN8          ------------------------------------------------------------
OsPIN6          ------------------------------------------------------------
AtPIN5          ------------------------------------------------------------
AtPIN6          ---------------IYRQSSPKM------------------------------------
PpPINC          -------SMGPDGRTIYPGITVVTNSVAA---------VPASGVSTHIINPVFSPLVSQV
PpPINA          LSTLGAPGMGPDGRTIYPGSQTAINILTLGGAANVNATAPSTAVNTQIVNPVYSPQASQI
PpPINB          LSTLGTPGMGPDGRTIYPGSQTAISLVTPGGTGNIATPL-SSSLNTQIVNPVYSPRASQI
OsPIN3B         ----------------RARASATV------------------------------------
AtPIN2          --------YNNNSVPSYPPPNPMFTGSTSGASGVKKKESGGGGSGGG-------------
OsPIN2          ----------------YPAPNPGMMPAPRKKELG--------------------------
AtPIN4          ----------------YPAPNPEFSTGTGVSTKPNKIPKENQQQLQEKD-----------
AtPIN7          ----------------YPAPNPEFSTGNKTGSKAPKENHHHVG-----------------
AtPIN3          ----------------YPAPNPEFSSTTTSTANKSVNKNPKDVNTNQQTTLPTGG-----
OsPIN3A         ----------------RPPKPPATTT----------------------------------
OsPIN1B         ------------------------------------------------------------
AtPIN1          -------GSGGGGGAHYPAPNPGMFSPNTGGGGGTAAKGNAPVVGG--------------
OsPIN1C         ----------------YPAPNPAMAAPPKPKKAANG------------------------
OsPIN1A         ----------------YPAPNPAVSSAPKGAKKAA-------------------------
                         S337

PpPIND          -------------------------------------------------------NGESG
OsPIN5          -------------------------------------------------------SGETT
OsPIN4          ------------------------VMSSSSPP-----------------------EKQSD
OsPIN5B         -------------------------------------------------------TDDDD
AtPIN8          -------------------------------------------------------NIESG
OsPIN6          -------------------------------------------------------AAGKD
AtPIN5          -------------------------------------------------------KEEED
AtPIN6          ------------------------MWESGQRHAAK----DINGSVPE--------KEISF
PpPINC          AKKVNDPRASIPKTDEEAKELHMFVSSANPTSVSE-GELHVFGGSDISINL----QQSVN
PpPINA          AKKVKDPKAS-PRADEDAKELHMFVWSANASPVSE-AGLHVFGGNDTSANL----QQRFD
PpPINB          AKKVKDTRTS-PKSDEDAKELHMFVWSANASPVSE-AGLHVFGGNDTSANL----HQSFD
OsPIN3B         ------------APTNDLKDTHMIEWSSGASAASEVTGLPVFRSGRETRRLVP--SDAPS
AtPIN2          -----------VGVGGQNKEMNMFVWSSSASPVSEANAKNAMTRGSS--------TDVST
OsPIN2          -----------GSNSNSNKELHMFVWSSSASPVSE---ANLRNAVNHAAST----DFASA
AtPIN4          -----------SKASHDAKELHMFVWSSSASPVSD-----VFGGGAGDNVATE--QSEQG
AtPIN7          -----------KSNSNDAKELHMFVWGSNGSPVSDRAGLQVDNGANEQVGK----SDQGG
AtPIN3          -----------KSNSHDAKELHMFVWSSNGSPVSDRAGLNVFGGAPDNDQGG---RSDQG
OsPIN3A         -----------GALNHDAKELHMFVWSSSASPVSEVSGLPVFSGGGGGG------ALDVG
OsPIN1B         -------------VVGKRKDLHMFVWSSSASPVSER-------------------AAAAA
AtPIN1          -----------KRQDGNGRDLHMFVWSSSASPVSD-----VFGGGGGNHHADYSTATNDH
OsPIN1C         -----------QAKGEDGKDLHMFVWSSSASPVSD-----VFGNGAEY-------NDAAA
OsPIN1A         -----------TNGQAKGEDLHMFVWSSSASPVSD-----VFGGGAPDY------NDAAA
                              S377

PpPIND          TRE------NGTEHGHE-------------------------------------------
OsPIN5          AAAKEVSSGEVAPVEEEEA-----------------------------------------
OsPIN4          VEMNGAVVAAPGGGGGVRL-----------------------------------------
OsPIN5B         VEDGAAAAATAAAA----------------------------------------------
AtPIN8          KRETVVV-----------------------------------------------------
OsPIN6          VEAAGAAAAAGTVVVAAAA-----------------------------------------
AtPIN5          EEEVAI------------------------------------------------------
AtPIN6          RDALKAAPQATAAGGGASM----------------E-EGAAGK-----------------
PpPINC          PKELHVHVHPQSEHHLPGAANHKTQ-----DEHARQ-GFSFGNRR----DLKVEDVDNNG
PpPINA          PKEVRMLVHPQLDRGLAAA-SPRTY-----DEYTRE-DFSFGNRN----DLKLEDLDKDG
PpPINB          PKEVRMLVHPQSDLRHPEA-NPRTY-----DNYAQE-DFSFGNRN----DLKLEDLDKDG
```

```
OsPIN3B         IASSRVIRPPPGATGGERA--------------ASF-NKAVGGQ---------DELAKLE
AtPIN2          DPKVSIPPHDNLATKAMQN--------------LIE-NMSPGRKG----HVEMDQDGNNG
OsPIN2          PPPAAVPVGGATPKGVSGSVTPAAK-----NGGGEL-EIEDG-------------LKSPA
AtPIN4          AKEIRMVVSDQPRKSNARG----GG-----DDIGGL-DSGEGER----------EIEKAT
AtPIN7          AKEIRMLISDHTQNGENKA--------------GPM-NGDYGG------EEESERVKEVP
AtPIN3          AKEIRMLVPDQSHNGETKAVAHPAS-----GDFGGEQQFSFAGK-----EEEAERPKDAE
OsPIN3A         AKEIHMVIPADLPQNNGSGKEHEEYGAVALGGGGGGENFSFGGGKTVDGAEAVDEEAALP
OsPIN1B         AGAVHVFGGGGADHGDAKG------------AQAYD-EYSFGNKN-----------EKDG
AtPIN1          QKDVKISVPQGNSNDNQYV--------------ERE-EFSFGNKDDDSKVLATD------
OsPIN1C         VKEVRMAVASPRKADGVER----------------D-DFSFGNRGVAERDAEAGDEKSVA
OsPIN1A         VKSPRKMDGAKDREDYVER----------------D-DFSFGNRG----VMDRDAEAGDE


PpPIND          ----------------------------------------MAPSQMNLKEMAIKVAKKMV
OsPIN5          ----------------------------------------SAPAPSMKHVIWMA-VKKLL
OsPIN4          ------------------------------------------PFWATARTV----GLKLA
OsPIN5B         ----------------------------------------RRSLWPLVRAV----WLKVA
AtPIN8          ---------GEKSFLE----------------------------------VMSLVWLKLA
OsPIN6          ---------G------------------------------KPSLWALVKVV----AHKLA
AtPIN5          ----------------------------------------VRTRSVGTMKILLKAWRKLI
AtPIN6          ---------DTTPVAAIGKQE-------------------MPSAIVMMRLILTVVGRKLS
PpPINC          SKLDKKF--RSILTAELAPKHPMDEG---KTS--------MPPSSVMIKLICVMTFRKLT
PpPINA          PRLDKF---GSTSTAELTPKLAEDEA---KKS--------MPPSAVMIKLIAVMTFRKLV
PpPINB          PRLDNKF--GSTSTAELTPKVPEDEA---KKS--------MPPSAVMIKLIAVMTFRKLV
OsPIN3B         AGAKTE---QQTTAVTTTTKGGGAAG---AER---ARGQQNAPAGVMLRLILTTVWRRLI
AtPIN2          ---------GKSPYMGKKGSDVEDGGPGPRKQQ-------MPPASVMTRLILIMVWRKLI
OsPIN2          AGLAAKFPVSGSPYVAPRKKGGGADV---PGL--AEAAHPMPPTSVMTRLILIMVWRKLI
AtPIN4          AGLNKM---GSNSTAELEAAGGDGGG---NNGTH------MPPTSVMTRLILIMVWRKLI
AtPIN7          NGLHKL---RCNSTAELNPKEAIETG---ETV----PVKHMPPASVMTRLILIMVWRKLI
AtPIN3          NGLNKL---APNSTAALQSKTGLGGAEASQRKN-------MPPASVMTRLILIMVWRKLI
OsPIN3A         DGLTKM---GSSSTAELHPKVVDVDGPNAGGGAAGAGQYQMPPASVMTRLILIMVWRKLI
OsPIN1B         PTLSKL---GSNSTAQLRPKDDGEGM---AAA--------MPPASVMTRLILIMVWRKLI
AtPIN1          ---------GGNNISNKTTQAKV-----------------MPPTSVMTRLILIMVWRKLI
OsPIN1C         AAVS-----GEHGKPGLTPAPTA-----------------MPPTSVMTRLILIMVWRKLI
OsPIN1A         KAAAAA---GADPSKAMAAPTA------------------MPPTSVMTRLILIMVWRKLI
                                                                  :      .:


PpPIND          QLPLTHATVMGIVYSLIAGRWGFDPLRILRNSLDIMGRITLGLTMYSIGLFMAGQKKLVA
OsPIN5          QIPNTYASFLGLIWSLIAFKCGFSMPKIVEDSLFTIRTTAVGLSMFSSGTFIARQSRFVP
OsPIN4          RNPNVYASVLGVVWACIAYRWHLSLPGIVTGSLQVMSRTGTGMSMFSMGLFMGQQERVIA
OsPIN5B         RNPNVYAGVLGVAWACVTNRWHVETPSIIEGSVLIMSKTGVGLSMFSMGLFMALQDKIIV
AtPIN8          TNPNCYSCILGIAWAFISNRWHLELPGILEGSILIMSKAGTGTAMFNMGIFMALQEKLIV
OsPIN6          RNPNTYASFVGITWACLANRLHIALPSAFEGSVLIMSKSGTGMAMFSMGLFMAQQEKIIA
AtPIN5          INPNTYATLIGIIWATLHFRLGWNLPEMIDKSIHLLSDGGLGMAMFSLGLFMASQSSIIA
AtPIN6          RNPNTYSSLLGLVWSLISFKWNIPMPNIVDFSIKIISDAGLGMAMFSLGLFMALQPKMIP
PpPINC          RNPNTYSSLLGVVWSLISFKCHLDMPLILYKSYHIISDAGIGMAMFSLGLFMGMGDRIIA
PpPINA          WNPNTYSSLLGVIWSLVANRWHLSMPLILYKSVHILSDAGLGMAMFSLGLFMGLGDRIIV
PpPINB          WNPNTYSSLLGVIWSLVANRWHFTMPLILYKSVHILSDAGLGMAMFSLGLFMGLGDRIVV
OsPIN3B         RNPNTYASLIGLTWSLIAFRFHITMPIIVAKSISILSDAGLGMAMFSLGLFMATQPKIIA
AtPIN2          RNPNTYSSLFGLAWSLVSFKWNIKMPTIMSGSISILSDAGLGMAMFSLGLFMALQPKIIA
OsPIN2          RNPNTYSSLIGLVWSLVSFRWNIQMPSIIKGSISILSDAGLGMAMFSLGLFMALQPKIIS
AtPIN4          RNPNTYSSLIGLIWALVAYRWHVAMPKILQQSISILSDAGLGMAMFSLGLFMALQPKIIA
AtPIN7          RNPNTYSSLIGLIWALVAFRWDVAMPKIIQQSISILSDAGLGMAMFSLGLFMALQPKLIA
AtPIN3          RNPNTYSSLIGLIWALVAFRWHVAMPKIIQQSISILSDAGLGMAMFSLGLFMALQPKLIA
OsPIN3A         RNPNTYSSLLGLAWSLVAFRWHVSMPAIVEKSISILSDAGLGMAMFSLGLFMALQPSIIA
OsPIN1B         RNPNTYSSLLGVIWSLVSYRWGIEMPAIIARSISILSDAGLGMAMFSLGLFMALQPRIIA
AtPIN1          RNPNSYSSLFGITWSLISFKWNIEMPALIAKSISILSDAGLGMAMFSLGLFMALNPRIIA
OsPIN1C         RNPNTYSSLIGLIWSLVCFRWNFEMPAIILKSISILSDAGLGMAMFSLGLFMALQPRIIA
OsPIN1A         RNPNTYSSLIGLIWSLVCFRWNFEMPAIVLKSISILSDAGLGMAMFSLGLFMALQPHIIA
```

```
                  *  :: ..*: :: :  .        .  *   :     * :*:. * *:.    .:

PpPIND          S-WWVAFYGAFCRFIVGPGTMGVASLLLGLRGDTLRFAFLQAALPQAVISFVFAKDYNLH
OsPIN5          CGYKIASFSMVIKFLIGPVVMLFASLVIGMHGTLLHIAVVQAALPLAVTSFVYAEEYKVH
OsPIN4          CGAGLTALGMALRFVAGPLATLVGAAALGLRGDVLHLAIIQAALPQSIASFVFAKEYGLH
OsPIN5B         CGAGLTVLGMALRFVAGPAATAVGAFALGLRGDLLRLAIIQAALPQSITTFVFAKEYGLH
AtPIN8          CGTSLTVMGMVLKFIAGPAAMAIGSIVLGLHGDVLRVAIIQAALPQSITSFIFAKEYGLH
OsPIN6          CGTSFAALGLVLKFALGPAAMAIGSIAVGLRGDVLRVAIIQAALPQSITSFIFAKEYGLH
AtPIN5          CGTKMAIITMLLKFVLGPALMIASAYCIRLKSTLFKVAILQAALPQGVVPFVFAKEYNLH
AtPIN6          CGAKKATMGMLIRFISGPLFMAGASLLVGLRGSRLHAAIVQAALPQGIVPFVFAREYNLH
PpPINC          CGTKHALFAMLLRFLVGPAVFAAASYLVGLRGVSLNVSTVQAALPQGIVPFVFAKEYNVH
PpPINA          CGTKMAVFGMALRFLAGPAVFAAASYLVGLRGVPLKVSIVQAALPQGIVPFVFAKEYGVH
PpPINB          CGRKMAIFGMSLRFLAGPAVFAAASYLVGLRGVPLKVSIVQAALPQGIVPFVFAKEYGVH
OsPIN3B         CGYSVAAASMGVRFFFGPAIMAAASAAVGIRGTLLRIAIVQAALPQGIVPFVFAKEYNLH
AtPIN2          CGKSVAGFAMAVRFLTGPAVIAATSIAIGIRGDLLHIAIVQAALPQGIVPFVFAKEYNVH
OsPIN2          CGKTVATFAMAVRFLTGPAVIAATSIAIGLRGVLLHVAIVQAALPQGIVPFVFAKEYNCH
AtPIN4          CGNSVATFAMAVRFITGPAIMAVAGIAIGLHGDLLRIAIVQAALPQGIVPFVFAKEYNVH
AtPIN7          CGNSTATFAMAVRFFTGPAVMAVAAMAIGLRGDLLRVAIVQAALPQGIVPFVFAKEYNVH
AtPIN3          CGNSVATFAMAVRFLTGPAVMAVAAIAIGLRGDLLRVAIVQAALPQGIVPFVFAKEYNVH
OsPIN3A         CGKSAAVVSMAVRFLAGPAVMAAASIAIGLRGTLLHVAIVQAALPQGIVPFVFAKEYNVH
OsPIN1B         CGNSLASYAMAVRFLVGPAVMAAASIAVGLRGVLLHIAIVQAALPQGIVPFVFAKEYNVH
AtPIN1          CGNRRAAFAAAMRFVVGPAVMLVASYAVGLRGVLLHVAIIQAALPQGIVPFVFAKEYNVH
OsPIN1C         CGNKVATFAMAVRFLTGPAVMAAASIAVGLRGTLLHVAIVQAALPQGIVPFVFAKEYSVH
OsPIN1A         CGNKVATYAMAVRFLAGPAVMAAASFAVGLRGTLLHVAIVQAALPQGIVPFVFAKEYSVH
                .    :      .*  **      .  : :..  :. : :***** .: .*::* :*  *

PpPIND          TDVFTTAVSLQTIVFMPIVLVYYTLLEL--
OsPIN5          ADIMSTGVILGIFISLPVTIVYYILLGL--
OsPIN4          ADVLSTAVIFGTLISLPILIAYYAVLGFV-
OsPIN5B         AEILSTAVIFGTLASLPVLIVYYIVLGFIR
AtPIN8          ADVLSTAVIFGMLVSLPVLVAYYAALEFIH
OsPIN6          ADVLSTAVIFGMLVSLPLLVGFYIVLELIR
AtPIN5          PEIISTGVIFGMLIALPTTLAYYFLLDL--
AtPIN6          PDLLSTLVIFGMIVSLPVTILYYVLLGL--
PpPINC          PEILSTAVIFGMIVTLPTALLYYILLGL--
PpPINA          PEILSTAVIFGMLIALPITMVYYILLGL--
PpPINB          PEMLSTAVIFGMLIALPITMVYYILLGL--
OsPIN3B         ATILCTFVIFGMLIALPITLVYYIILGL--
AtPIN2          PDILSTAVIFGMLVALPVTVLYYVLLGL--
OsPIN2          PQILSTAVIFGMLIALPITILYYVLLGI--
AtPIN4          PTILSTGVIFGMLIALPITLVYYILLGL--
AtPIN7          PAILSTGVIFGMLIALPITLVYYILLGL--
AtPIN3          PAILSTGVIFGMLIALPITLVYYILLGL--
OsPIN3A         PAILSTAVIFGMLIALPITLLYYILLGL--
OsPIN1B         PNILSTAVIFGMLIALPITLVYYILLGL--
AtPIN1          PDILSTAVIFGMLIALPITLLYYILLGL--
OsPIN1C         PDILSTAVIFGMLIALPITLVYYILLGL--
OsPIN1A         PSILSTAVIFGMLIALPITLVYYILLGL--
                . :: * * :  :  :*  : :*  * :
```

Supp. Fig. 1. Sequence alignment of Arabidopsis, rice and *Physcomitrella patens* members of the UNIPROT PIN auxin efflux protein family. Multiple sequence alignment was performed by the MUSCLE algorithm (v3.8). Potential MAPK phosphorylation sites (S/TP motifs) and their positions in PIN1 are indicated underneath with red font. MAPK and PID phosphorylation sites and preferred MAPK phosphorylation flanking residues are highlighted in red, black and grey backgrounds, respectively.

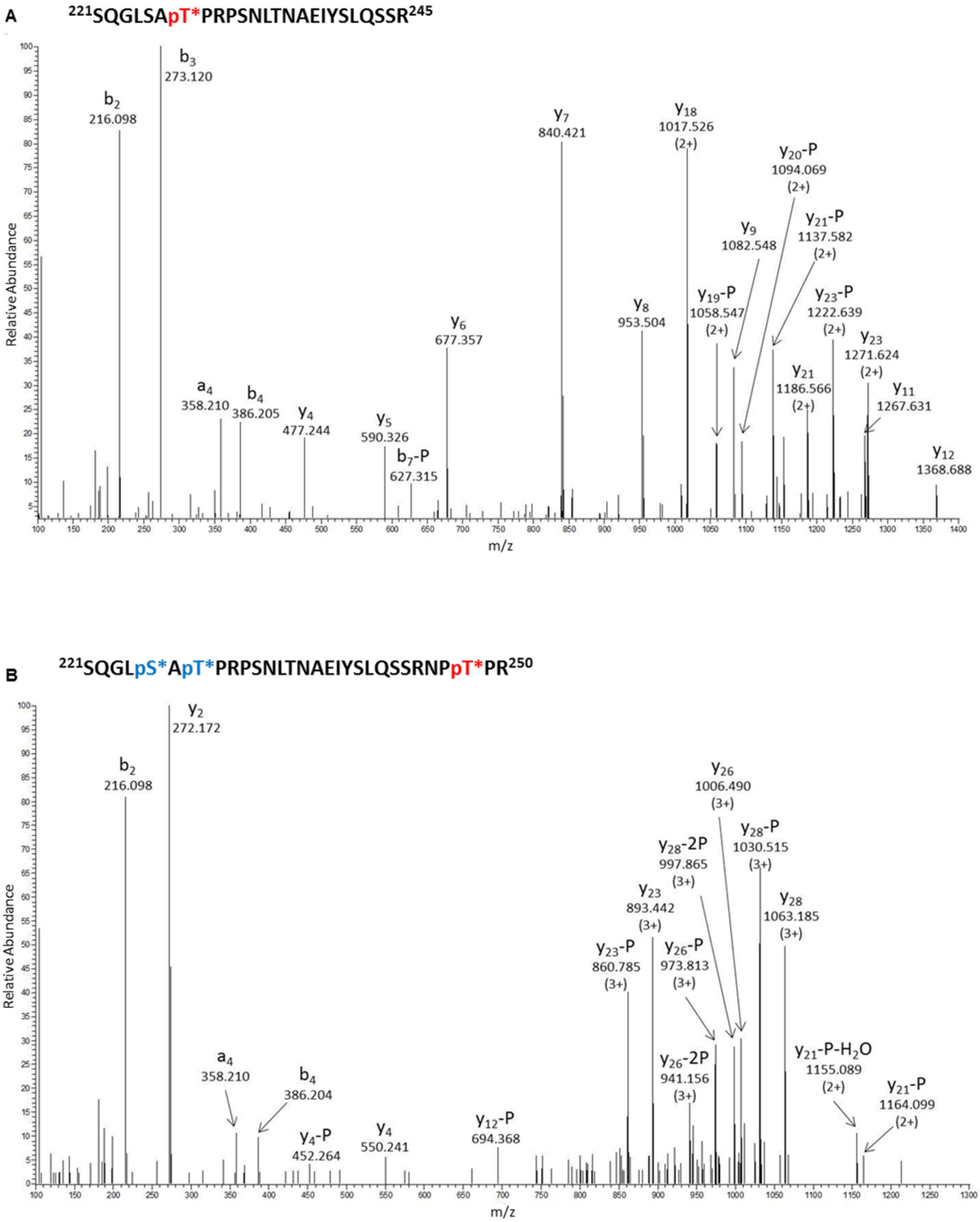
A
221SQGLSApT*PRPSNLTNAEIYSLQSSR245
Relative Abundance
m/z
b2 216.098
b3 273.120
a4 358.210
b4 386.205
y4 477.244
y5 590.326
b7-P 627.315
y6 677.357
y7 840.421
y8 953.504
y18 1017.526 (2+)
y19-P 1058.547 (2+)
y9 1082.548
y20-P 1094.069 (2+)
y21-P 1137.582 (2+)
y21 1186.566 (2+)
y23-P 1222.639 (2+)
y23 1271.624 (2+)
y11 1267.631
y12 1368.688
B
221SQGLpS*ApT*PRPSNLTNAEIYSLQSSRNPpT*PR250
Relative Abundance
m/z
b2 216.098
y2 272.172
a4 358.210
b4 386.204
y4-P 452.264
y4 550.241
y12-P 694.368
y23-P 860.785 (3+)
y23 893.442 (3+)
y26-2P 941.156 (3+)
y26-P 973.813 (3+)
y28-2P 997.865 (3+)
y26 1006.490 (3+)
y28-P 1030.515 (3+)
y28 1063.185 (3+)
y21-P-H2O 1155.089 (2+)
y21-P 1164.099 (2+)

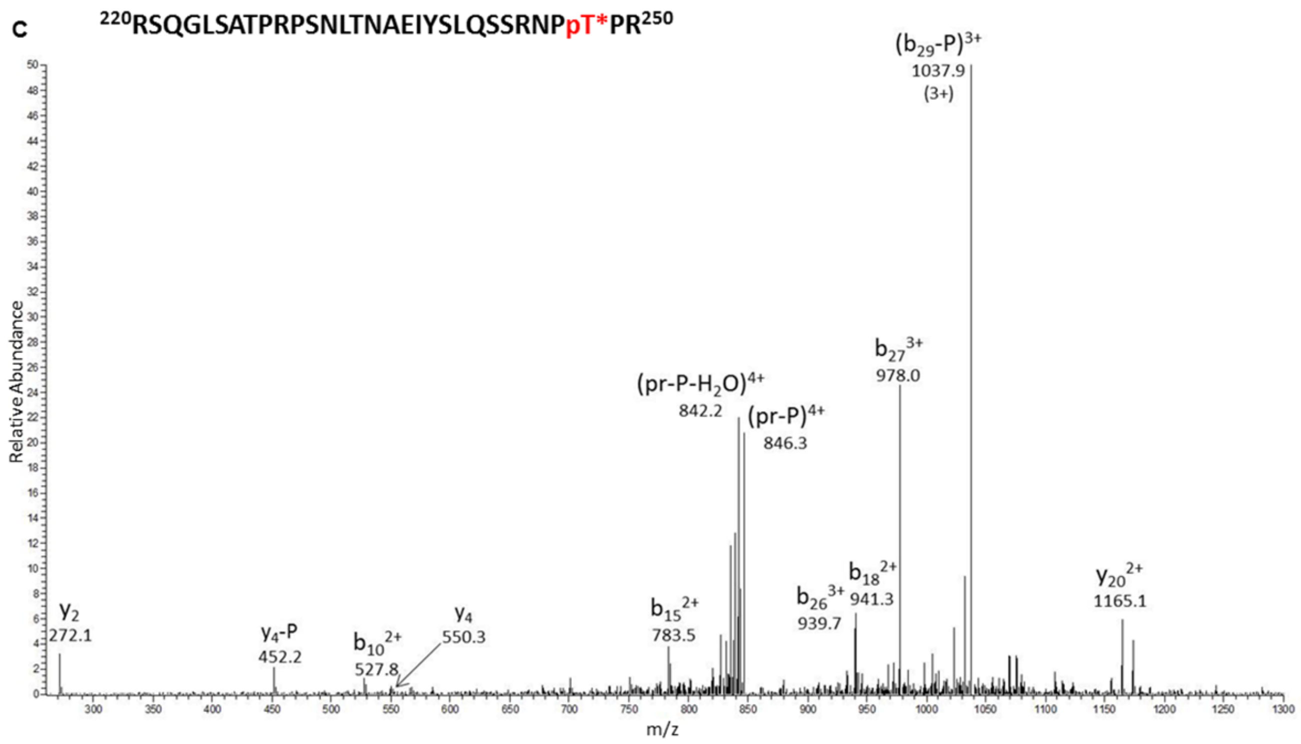

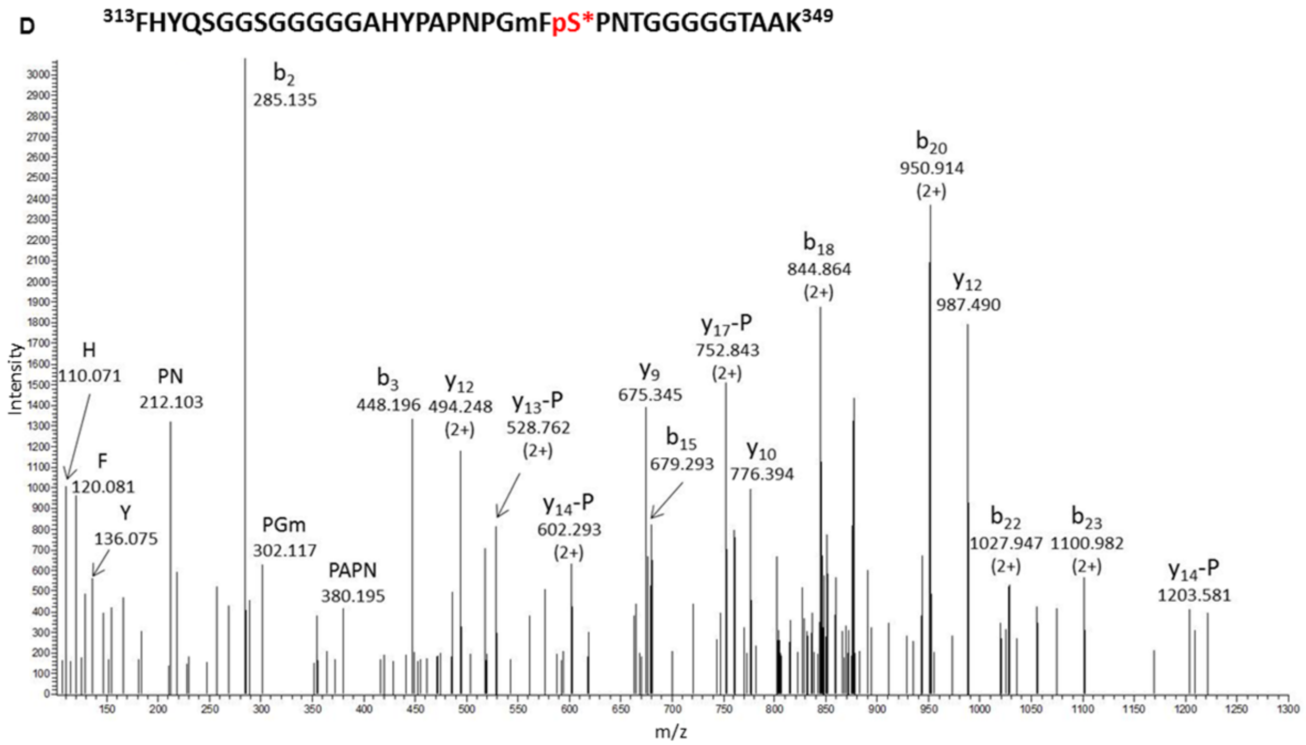

Supp. Fig. 2. Spectra for representative identified phosphopeptides derived from PIN1 hydrophilic loop. HCD spectrum representing T286 phosphorylation is presented as Fig. 4C in the main text.

(A-D) Sequence of the precursor peptides are indicated above the spectra. Asterisk indicates phosphorylated Serine/Threonine. Peptide fragments are labelled according to the

nomenclature by Biemann (1990). -P stands for the 98-Da neutral loss of phosphoric acid characteristic to Ser/Thr phosphorylation. (A) HCD spectrum of m/z: 919.7788 (3+) representing SQGLSApTPRPSNLTNAEIYSLQSSR, [221-245] of PIN1. Site of phosphorylation is Thr-227 as proven by unmodified y_{18} and y_{19}-P fragment ions (m/z 1108.1 representing y_{19} was observed in the CID spectrum of the same precursor, data not shown). (B) HCD spectrum of m/z: 851.400 (4+) representing the doubly phosphorylated SQGLSATPRPSNLTNAEIYSLQSSRNPTPR, [221-250] of PIN1. One phosphorylation site is Thr-248 as proven by the y_4 fragment ion. The other site is either Ser-225 or Thr-227 proven by the (singly phosphorylated) y_{23} and the (doubly phosphorylated) y_{26} fragment ions (indicated by blue font). (C) CID spectrum of m/z: 870.4335 (4+) representing RSQGLSATPRPSNLTNAEIYSLQSSRNPpTPR, [220-250] of PIN1. Site of phosphorylation is Thr-248 as proven by y_4 and b_{27} fragment ions. (D) HCD spectrum of m/z: 876.1229 (4+) representing FHYQSGGSGGGGGAHYPAPNPGmFpSPNTGGGGGTAAK,
[313-349] of PIN1. m denotes oxidised Met. Site of phosphorylation is Ser-337 as proven by unmodified y_{12} and b_{23} fragment ions.

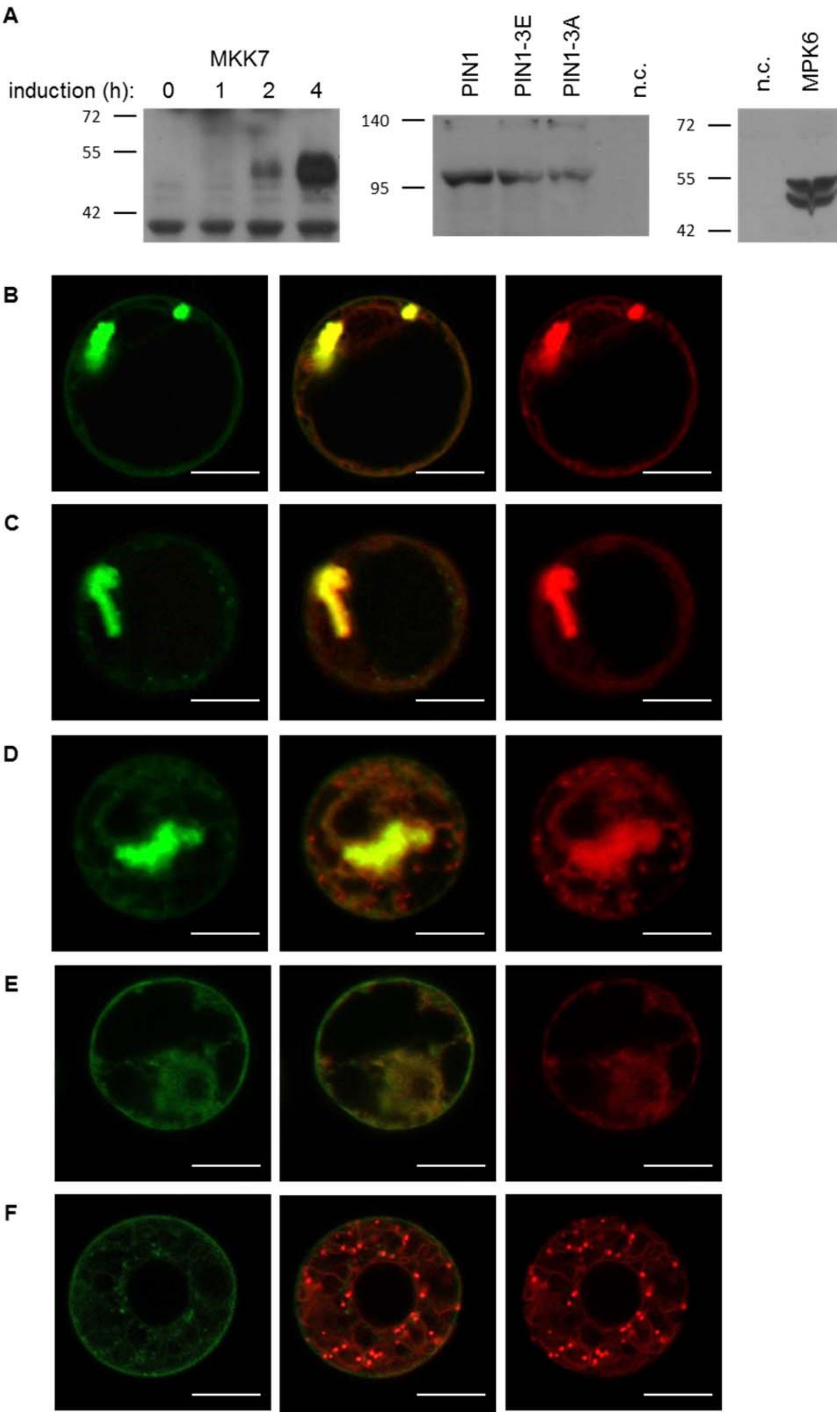
A
MKK7
induction (h): 0 1 2 4
72
55
42
PIN1
PIN1-3E
PIN1-3A
n.c.
140
95
n.c.
MPK6
72
55
42
B
C
D
E
F

Supp. Fig. 3. MAPK phosphorylation status at T227, T248 and T286 influences intracellular trafficking of PIN1. (A) Immunodetection of transfected proteins. PIN1:GFP MPK6:HA and myc:MKK7 were detected by commercial antibodies recognising the respective fusion tags. n.c. denotes non-transformed control. Molecular weight (kDa) markers are indicated. Protoplasts were transformed with all constructs simultaneously, rested overnight then treated with ß-estradiol for 4h if transfected with pER8:MKK7 prior to protein isolation or microscopy. (B-F) Co-localisation of PIN1 variants of differential phosphorylation status (green, left panels) with intracellular organelle markers (Nelson et al., 2007) (red, right panels) and merge images (middle panels). (B) PIN1 co-transformed with MPK6 and *pER8:MKK7*, induced with 1 µM β-estradiol for 4h with RFP:ER marker. (C) Phosphomimetic T227E, T248E, T286E (PIN1-3E) PIN1 mutant version with RFP:ER marker. (D) PIN1-3E with RFP:Golgi marker. (E) Non-phosphorylatable T227A, T248A, T286A (PIN1-3A) with RFP:ER marker. (F) PIN1-3A with RFP:Golgi marker. Scale bar: 10 µm.
